# The Urokinase-Type Plasminogen Activator Receptor (uPAR) as a Mediator of Physiological and Pathological Processes: Potential Therapeutic Strategies

**DOI:** 10.3390/cancers17203309

**Published:** 2025-10-14

**Authors:** Ali Iftikhar, Niaz Mahmood, Shafaat A. Rabbani

**Affiliations:** 1 Department of Human Genetics, McGill University, Montréal, QC H4A 3J1, Canada; 2 Department of Medicine, McGill University, Montréal, QC H4A 3J1, Canada

**Keywords:** urokinase, receptor, cancer, metastasis, cell signaling

## Abstract

The urokinase-type plasminogen activator receptor (uPAR) plays a pivotal role in regulating extracellular proteolysis, cell migration, immune responses, and tissue remodeling across diverse physiological and pathological contexts. This review provides detailed insights into the structure of uPAR, ligand interactions, and signaling mechanisms, emphasizing its central function in cancer progression, including tumor invasion, metastasis, angiogenesis, and modulation of the tumor microenvironment. We also summarize the involvement of uPAR as a key player in cardiovascular, infectious, and neurological diseases, where it contributes to inflammation, tissue damage, and disease progression. However, translational gaps remain, most notably inconsistent assay harmonization (especially for suPAR), uncertain context-specific cut-offs and patient-selection criteria and limited multicenter validation for uPAR-targeted imaging and therapeutics. This review addresses these gaps by synthesizing cross-disease evidence to clarify clinical use cases and outline practical selection frameworks. Furthermore, we discuss the clinical potential of uPAR as a diagnostic and prognostic biomarker in diverse disease contexts, along with recent advances in therapeutic strategies targeting uPAR.

## Simple Summary

uPAR (*PLAUR*) is a GPI-anchored receptor that concentrates pericellular proteolysis and couples it to cell migration and invasion via urokinase (uPA), vitronectin, integrins and the epidermal growth factor receptor (EGFR). These complexes activate FAK/Src-ERK and PI3K-AKT signaling, promoting the epithelial-to-mesenchymal transition, angiogenesis, immune modulation and metastatic dissemination. The circulating form, suPAR, is quantified in blood and often tracks disease activity. This review provides a detailed synthesis of uPAR biology across oncology and selected cardiovascular, infectious and neurological settings and evaluates diagnostics (including uPAR-targeted imaging) and therapeutics: uPA–uPAR antagonists (peptides/small molecules) and anti-uPAR antibodies that disrupt uPAR–integrin signaling (e.g., huATN-658) and RNA-based approaches.

## 1. Introduction

The urokinase plasminogen activator receptor (uPAR), also known as cluster of differentiation 87 (CD87), is a glycosylphosphatidylinositol (GPI)-anchored cell surface receptor that plays a crucial role in various physiological and pathological processes [1,2]. uPAR is integral to the regulation of proteolytic activity, cell migration, and tissue remodeling, which are vital for processes such as wound healing, inflammation, and cancer metastasis [3,4,5,6,7,8,9,10]. Mechanistically, uPAR binds urokinase-type plasminogen activator (uPA) to convert plasminogen to plasmin resulting in the activation of matrix metalloproteinase activation at the cell surface, while cooperating with integrins (α5β1, αvβ3) and the epidermal growth factor receptor (EGFR) to trigger FAK/Src and PI3K–AKT/ERK signaling that drives directed migration and tissue remodeling [3,4,5,6,7,8,9,10].

**Figure 1:**
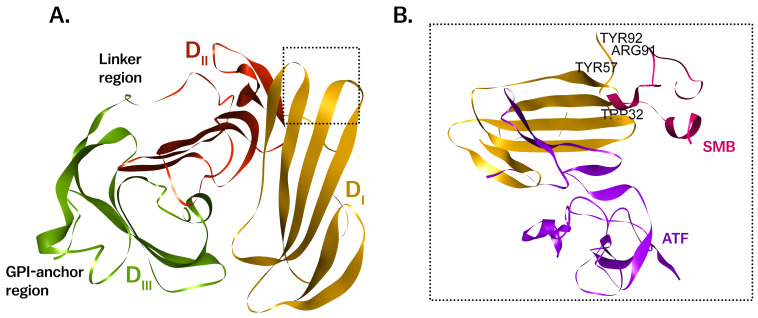
Crystal view of the ternary uPA–uPAR–vitronectin (VN) complex (PDB: 3BT1). (**A**) uPAR is a three-domain, GPI-anchored receptor composed of Ly6/uPAR modules DI (orange), DII (red), and DIII (green) connected by a flexible linker; the GPI-anchor region is indicated. The uPA-binding cavity lies at the DI–DII interface. (**B**) Detail of the DI surface showing the uPA amino-terminal fragment (ATF, purple) and the somatomedin B-like (SMB) domain of vitronectin (pink) docked on DI; representative DI residues (e.g., Trp32, Tyr57, Arg91, Tyr92) stabilize these contacts. Functional significance: concurrent binding of ATF and SMB creates a high-affinity ternary complex that orients pericellular plasminogen activation at the cell surface and stabilizes uPAR–matrix interactions, thereby coupling localized proteolysis with adhesion/signaling via integrin partners. Abbreviations: ATF, amino-terminal fragment of uPA; SMB, somatomedin B-like domain; VN, vitronectin.

The human uPAR is encoded by the *PLAUR* gene, located on chromosome 19q13.31. The canonical *PLAUR* gene consists of seven exons, encoding a 335-amino acid polypeptide that undergoes post-translational modifications, including glycosylation, to form the mature receptor [11,12]. Structurally, uPAR is composed of three homologous domains (DI, DII, and DIII) connected by two flexible hinges that collectively form a concave surface to interact with its ligand, urokinase plasminogen activator (uPA) (Figure 1). uPA is a serine protease that plays a critical role in converting inactive zymogen plasminogen into active plasmin, a potent enzyme responsible for degrading the components of the extracellular matrix (ECM). This proteolytic activity is essential for processes like cell migration, invasion, and tissue remodeling [13]. Additionally, uPAR interacts with other cell surface proteins, including integrins, vitronectin, and the epidermal growth factor receptor (EGFR) [14]. These interactions not only regulate proteolytic activity but also modulate cell adhesion, migration, and signal transduction pathways that are essential for maintaining tissue homeostasis [13,15].

### Role of uPAR in Physiology and Pathology

In normal physiology, uPAR plays a vital role in tissue remodeling, immune surveillance, and wound healing by localizing proteolytic activity to the cell surface through its interaction with uPA [13,16]. This interaction facilitates the conversion of plasminogen to plasmin [17,18,19,20]. By mediating the controlled breakdown of ECM components, such as fibrin and fibronectin, uPAR enables the migration of cells, including fibroblasts and immune cells, to sites of injury or inflammation, supporting effective tissue regeneration [21,22,23]. In parallel, the uPAR augments innate immune surveillance by promoting neutrophil and macrophage recruitment and chemotaxis to sites of injury or infection, helping maintain a balanced inflammatory response [24,25]. Through its interactions with integrins, uPAR is also involved in vascular homeostasis and angiogenesis, contributing to the stabilization of new blood vessels and maintaining proper tissue perfusion [26,27,28]. uPAR’s role in fibrinolysis, where it helps regulate the dissolution of blood clots, is also crucial in maintaining normal blood flow and preventing excessive clot formation. Through these multifaceted roles, uPAR ensures that proteolytic activity is tightly regulated at the cell surface, which is fundamental to maintaining tissue homeostasis and supporting physiological processes like wound healing, immune response, and vascular remodeling. However, in pathological conditions, particularly in cancer, uPAR expression is often dysregulated [5]. Elevated uPAR levels have been correlated with poor prognosis in several cancers, including breast, prostate, melanoma and colorectal cancers, where it enhances tumor invasiveness and metastatic potential [14,29,30,31,32,33,34,35,36,37,38,39].

uPAR is involved in chronic inflammatory and cardiovascular diseases, with clinical and experimental evidence implicating it in atherosclerosis and chronic obstructive pulmonary disease (COPD) via leukocyte recruitment, matrix metalloproteinase activation, and extracellular-matrix remodeling [40,41,42,43]. In atherosclerosis, uPAR-dependent signaling aligns with plaque inflammation and remodeling, while in COPD it associates with neutrophilic inflammation and airway structural change [3,44]. Reflecting this biology, circulating soluble uPAR (suPAR) levels correlate with disease activity and adverse outcomes across these conditions, albeit with context-dependent specificity.

Small molecule inhibitors, monoclonal antibodies, and RNA-based therapies targeting uPAR are currently under investigation, demonstrating promising preclinical and clinical outcomes in various cancers [45].

## 2. Molecular Structure of uPAR

### 2.1. Structural Domains and Ligand Binding

The protein structure of human uPAR consists of three homologous domains: DI, DII, and DIII, which are structurally connected to form a compact, ligand-binding surface. This modular structure enables uPAR to interact with its primary ligand, uPA, and other binding partners involved in cell signaling and ECM degradation [3,13,46] (Figure 1A).

Beyond the static view provided by crystallography [47,48,49,50,51], uPAR is known to exhibit significant conformational flexibility, which is vital for its interactions with various co-receptors and its role in cell signaling. The DI domain of uPAR directs uPA activity to the cell surface through its binding to the growth factor-like domain (GFD) of uPA [52].

Each of the three domains of uPAR is characterized by a Ly6/uPAR/α-neurotoxin (LU) domain, which contributes to the receptor’s ability to bind its ligand, uPA [53]. The DI domain is particularly crucial for binding to the growth factor-like domain (GFD) of uPA. The interaction between uPAR and uPA is highly specific and involves a deep hydrophobic pocket within the D1 domain, which accommodates the GFD of uPA [54].

**Figure 2:**
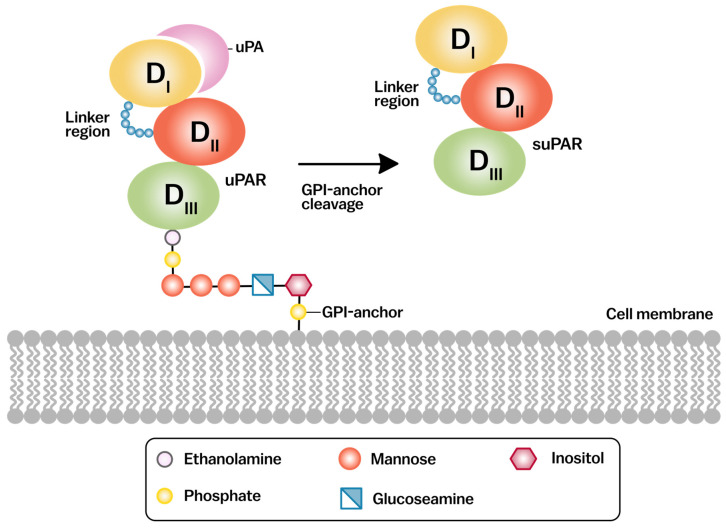
Schematic representation of the urokinase receptor. The uPAR is composed of three disulfide-bonded domains (DI, DII, DIII) and is anchored to the cell surface via GPI linkage. Domain DI forms the composite binding site for the uPA and DIII contribute to stabilizing the interaction between DI and uPA. Upon cleavage of the GPI anchor, suPAR is released into circulation. Both uPAR and suPAR can undergo further cleavage between domains DI and DII to generate the DI and DII-DIII fragments, the latter of which exhibits direct chemotactic activity. uPA, urokinase-type plasminogen activator; uPAR, urokinase-type plasminogen activator receptor; suPAR, soluble uPAR; DI/DII/DIII, uPAR domains I–III; GPI, glycosylphosphatidylinositol; ECM, extracellular matrix.

The crystal structure of uPAR bound to uPA reveals that the interaction is primarily mediated by the insertion of a protruding β-hairpin of the GFD into the central cavity of the D1 domain. This binding is stabilized by several key residues within the DI domain, including Tyr57, Tyr92, and Arg91 and Trp32 which form critical hydrophobic contacts with uPA [53,55,56,57,58] (Figure 1). The DII and DIII domains, while not directly involved in uPA binding, are essential for maintaining the overall structural integrity of uPAR and for mediating interactions with other cell surface molecules such as integrins and vitronectin. Upon binding with uPA, uPAR can undergo proteolytic cleavage, releasing a soluble form of the receptor (suPAR) that exhibits context-dependent biological activity, particularly in immune signaling. and circulates in the bloodstream [59,60] (Figure 2). This flexibility is also crucial for stabilizing the uPAR-integrin complexes that mediate cell adhesion and migration [5,61].

### 2.2. uPAR in Cell Signaling Pathways

**Figure 3:**
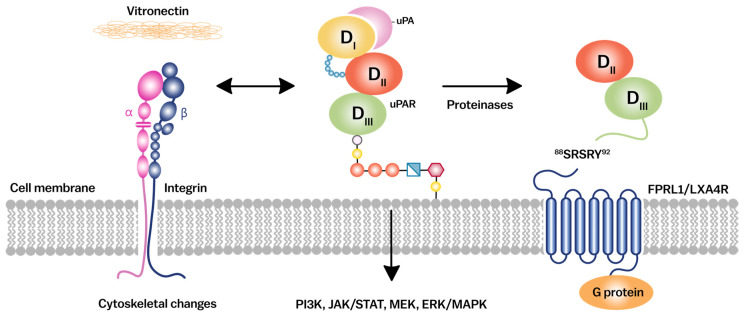
uPAR interacts with integrins and G-proteins to mediate downstream signaling pathways. Schematic diagram illustrates the central role of uPAR in signal transduction. uPAR interacts with integrins and G-protein–coupled receptors at the cell surface, facilitating the activation of multiple downstream pathways, including PI3K/AKT, JAK/STAT, and MAPK/ERK. These signaling cascades regulate diverse cellular processes such as proliferation, migration, and survival. VN, vitronectin; GPCR, G-protein–coupled receptor; FPRL1/LXA4R, formyl peptide receptor-like 1/lipoxin A4 receptor; PI3K, phosphoinositide 3-kinase; JAK/STAT, Janus kinase/signal transducer and activator of transcription; MEK, MAPK/ERK kinase; ERK/MAPK, extracellular signal-regulated kinase/mitogen-activated protein kinase.

uPAR mediates its effects primarily through its interactions with uPA and other co-receptors, such as integrins and G-protein-coupled receptors (GPCRs) [62] (Figure 3). Upon binding with uPA, uPAR undergoes a conformational change that allows it to associate with these co-receptors, thereby initiating a cascade of intracellular signaling events. These events include activation of the mitogen-activated protein kinase/extracellular signal-regulated kinase (MAPK/ERK), Janus kinase/signal transducer and activator of transcription (JAK/STAT), phosphoinositide 3-kinase–AKT/protein kinase B (PI3K/AKT), and nuclear factor kappa-B (NF-κB) pathways, which regulate gene expression, cytoskeletal rearrangement, and cell survival [63,64,65]. uPAR promotes metastatic spread by localizing plasminogen activation at the tumor–stroma interface, enabling ECM degradation and intravasation [24]. The interaction of uPAR with integrins is a central mechanism through which it modulates cell adhesion and migration (Section 3.2). Integrins are transmembrane receptors that facilitate ECM adhesion. uPAR can bind directly to integrins such as α5β1 and αvβ3, promoting the formation of focal adhesion complexes, which are essential for cell motility [24] (Table 1). These interactions are particularly important in cancer metastasis, where uPAR-integrin complexes facilitate the detachment of cancer cells from the primary tumor, their invasion through the ECM, and their eventual migration to distant sites [66,67,68,69]. uPAR also interacts with other cell surface receptors, including GPCRs and EGFR, enhancing the complexity and diversity of its signaling outcomes. The crosstalk between uPAR and these receptors can amplify signaling pathways, thereby enhancing cellular responses to external stimuli [14,70]. In cancer, the overexpression of uPAR and its interaction with these receptors can lead to aberrant activation of signaling pathways, contributing to uncontrolled cell proliferation, survival, and metastasis. Targeting these interactions presents a promising therapeutic strategy to inhibit uPAR-mediated signaling in cancer and other diseases [71]. A detailed list of uPAR binding ligands and their functional consequences is summarized in Table 1.

One critical pathway activated by uPAR is the MAPK/ERK signaling pathway. This pathway is initiated when uPAR interacts with integrins, leading to the activation of focal adhesion kinase (FAK) and Src family kinases. This subsequently triggers the Ras/Raf/MEK/ERK signaling cascade [10] which is involved in both the transcription and translation of mRNAs that promote cell proliferation and survival.

The PI3K/AKT pathway, involved in cell survival and anti-apoptotic functions [72,73], is another key pathway influenced by uPAR signaling [74,75]. When uPAR engages with integrins and GPCRs, it leads to the activation of PI3K, which converts phosphatidylinositol 4,5-bisphosphate (PIP2) to phosphatidylinositol 3,4,5-trisphosphate (PIP3), subsequently activating AKT. Activated AKT phosphorylates several downstream targets, such as Bad, FOXO transcription factors, and GSK-3β, that inhibit apoptotic pathways and promote cell survival [73,76,77].

Additionally, uPAR is involved in the regulation of the JAK/STAT signaling pathway, particularly in the context of inflammation and immune responses [78,79]. For example, pro-inflammatory cytokines such as interleukin-6 (IL-6) and tumor necrosis factor-alpha (TNF-α) have been shown to upregulate uPAR, as well as uPA secretion, in various cell types such as monocytes, endothelial cells, and tumor cells [80], which in turn can facilitate JAK1/2 activation and STAT3 phosphorylation, thereby amplifying inflammatory signaling cascades. Phosphorylated STAT dimerizes and translocate to the nucleus, where they induce the transcription of genes. encoding c-Myc, Cyclin D1, and Bcl-2, which are involved in cell proliferation, differentiation, and immune responses [79,81].

**Table 1:** Comprehensive List of uPAR Ligands and Interacting Partners in Cancer Biology.

Ligand/Interacting Partner	Binding Domain on uPAR	Functional Effects	Reference
uPA (urokinase-type plasminogen activator)	DI	Activates plasminogen, leading to ECM degradation, cell migration, and invasion	[82]
Vitronectin	DI, DII, DIII	Stabilizes the uPA-uPAR complex, enhances cell adhesion and migration	[61,83]
Integrins (e.g., α5β1, αvβ3)	DI, DII, DIII	Promotes focal adhesion formation, facilitates cell migration, signaling	[63,84,85]
Factor XII	DII	Initiates the intrinsic coagulation pathway and contributes to fibrinolysis and inflammatory responses	[86,87]
High molecular weight kinin-free kininogen (HKa)	DI-DII	Regulates inflammation and coagulation processes	[88]
Factor VIIa	DII	Involved in the extrinsic coagulation pathway, influences cell signaling	[86]
Tissue Factor Pathway Inhibitor (TFPI)	-	Regulate coagulation by inhibiting the TF-FVIIa complex	[89]
Thrombospondin-1 (TSP-1)	-	Inhibits angiogenesis, modulates uPAR-dependent signaling	[90,91]
Urokinase receptor-associated protein (uPARAP/Endo180)	DI-DIII	Facilitates collagen degradation, linked to ECM remodeling and tumor progression	[92]
Plasminogen	DI	Precursor of plasmin, plays a role in fibrinolysis, linked to cell migration	[93,94]
SRPX2	DI-DII-DIII	SRPX2 binds to uPAR, facilitating angiogenesis and tumor cell migration	[95]
Amino-terminal fragment (ATF)	DI	ATF binds to uPAR, promoting proteolysis, cell migration, and cancer progression	[45,88]
Cathepsin B	DI-DIII	Protease that can degrade ECM, involved in tumor progression and metastasis	[96]
Streptococcal surface dehydrogenase (SDH)	DI	SDH binds TO uPAR, aiding bacterial adherence and contributing to Group A *Streptococcus* infection	[97]
RAGE	-	RAGE interacts with uPAR through integrin αVβ3, promoting ROS production and tumor cell migration	[98]
uPAR-associated GPI-anchored proteins (GPI-APs)	GPI-anchor region	uPAR interacts with various GPI-APs, contributing to signal transduction and cell migration	[99]
α2-Macroglobulin	DI	Binds uPAR-uPA complex, involved in cell signaling and tissue remodeling	[100]
Fibroblast Activation Protein (FAP)	-	FAP interacts with uPAR through FAK-Src-JAK2 signaling, promoting tumor invasion and immune suppression	[101,102,103]
Complement component C3a	-	Involved in immune response modulation, enhances inflammation	[104]
gp130	-	gp130 interacts with uPAR via the JAK/STAT3 pathway, promoting tumor cell proliferation and immune modulation	[78]
EGFR (Epidermal Growth Factor Receptor)	DII-DIII	uPAR-EGFR interaction enhances EGFR signaling, leading to increased cell proliferation, migration, and survival	[52,105]
GPCRs (e.g., FPRL1/LXA4R)	Variable	Facilitates chemotaxis, immune response modulation, and cancer cell invasion	[52,106]

## 3. uPAR and Cancer

**Figure 4:**
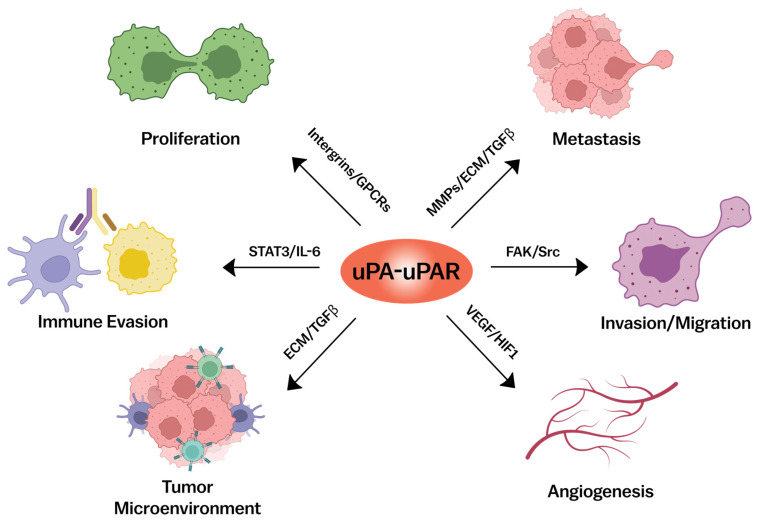
Schematic overview of the multifaceted roles of the uPA-uPAR system in cancer progression. The interaction between uPA and its receptor uPAR contributes to key tumor-promoting processes mediated through diverse signaling pathways: integrins and GPCRs promote proliferation; MMPs, ECM remodeling, and TGFβ facilitate metastasis; FAK/Src signaling enhances invasion and migration; VEGF and HIF1 drive angiogenesis; ECM and TGFβ contribute to shaping the tumor microenvironment; and STAT3/IL-6 signaling supports immune evasion. MMPs, matrix metalloproteinases; ECM, extracellular matrix; TGFβ, transforming growth factor beta; FAK/Src, focal adhesion kinase/Src family kinases; VEGF, vascular endothelial growth factor; HIF1, hypoxia-inducible factor-1; STAT3, signal transducer and activator of transcription 3; IL-6, interleukin-6; GPCRs, G-protein–coupled receptors.

uPAR’s activity is not confined to the cancer cells themselves but extends to the tumor microenvironment (TME), where it contributes to a pro-tumorigenic milieu [107,108] (Figure 4). In this section, we dissect the complex roles of uPAR in tumor progression, invasion, metastasis, angiogenesis and modulation of TME, highlighting its mechanisms of action and potential as a therapeutic target.

### 3.1. Involvement in Tumor Progression

uPAR-mediated activation of the PI3K/AKT pathway significantly enhances tumor cell survival by inhibiting apoptotic signals and promoting cellular proliferation under hypoxic conditions, common in solid tumors [31,73]. This pathway also contributes to the EMT, a process that endows epithelial cells with increased motility and invasiveness, further driving tumor progression. EMT is driven by the activation of transcription factors such as Snail, Twist, and ZEB1, which are upregulated in response to uPAR signaling. This transition is a key step in the metastatic cascade, enabling tumor cells to detach from the primary tumor and invade distant tissues [109].

### 3.2. Invasion and Metastasis

Invasion and metastasis are hallmark capabilities of cancer that distinguish it from benign growth. uPAR’s involvement in these processes is particularly critical. uPAR associates with α5β1 and αvβ3 integrins to form multiprotein adhesion complexes that activate FAK and Src, triggering downstream Ras–MAPK and PI3K–Akt signaling essential for cell motility and ECM engagement [34,110]. These signaling cascades also stimulate small GTPases Rac1 and Cdc42, enabling actin polymerization dynamics that support cell protrusion and movement. In particular, uPAR-integrin–mediated activation of Rac1/Cdc42 underlies the formation and maintenance of invadopodia, the actin-rich, membrane-based structures essential for ECM degradation and tumor cell invasion [111,112]. Moreover, uPAR enhances invasive behavior through induction of matrix metalloproteinases (especially MMP-2 and MMP-9) via the uPA/uPAR system, leading to focal pericellular proteolysis vital for breaking through basement membranes and establishing metastatic niches [110].

uPAR actively orchestrates cellular invasion and metastatic dissemination through multiple molecular mechanisms. Functional studies in metastatic MDA-MB-231 breast cancer models demonstrate that uPAR–β1 integrin complexes are present in vivo and that disruption of this interaction, via administration of a blocking peptide (p25), significantly impairs tumor progression and bone metastasis in xenograft models, confirming the essential role of uPAR in integrin-mediated adhesion and dissemination [68]. Concurrently, research in breast carcinoma cell lines shows that co-expression of uPAR and integrin αvβ3 activates the FAK–SRC–ERK2–FRA-1 signaling axis, which enhances invadopodia formation and ECM invasion, and correlates with poor clinical prognosis in patient samples [113].

### 3.3. uPAR in Bone Metastasis and Skeletal Remodeling

A series of studies have described the role of uPAR in breast and other common cancers where uPAR plays a key role in tumor metastasis to non-skeletal and skeletal sites [4,5,114,115,116,117]. Breast cancer frequently metastasizes to bone, affecting over 70% of patients with advanced disease, and bone metastases account for a disproportionate share of morbidity due to skeletal-related events (SREs) such as fractures, spinal compression, and hypercalcemia [114]. Bone metastases predominantly occur in highly vascular trabecular bone, where tumor cells exploit a network of growth factors, cytokines, proteases, and vitronectin-binding integrins to establish and expand metastatic niches [115].

Both osteoclasts and osteoblasts in the metastatic bone microenvironment express uPA and uPAR. Functional studies, including uPAR knockout models, demonstrate reduced osteoclast numbers and preserved bone mineral density, underscoring uPAR’s direct role in osteolytic activity and bone remodeling [116,117]. Mechanistically, uPAR forms functional complexes with vitronectin-binding integrin αvβ3 on osteoclasts and tumor cells, coordinating with Src-family kinases to activate downstream signaling (e.g., c-Src, Pyk2, p130Cas) that enhances osteoclast differentiation, extracellular matrix degradation, and tumor cell colonization in bone metastasis models [118].

In a previous study by our group using preclinical MDA-MB-231 TNBC bone metastasis model, the humanized anti-uPAR antibody huATN-658 significantly inhibited primary tumor growth and bone lesion formation [119]. When combined with the bisphosphonate zoledronic acid (Zometa), the antitumor and anti-osteolytic effects were even more pronounced, featuring preserved bone architecture, decreased osteoclast prevalence (~95% vs. control/monotherapy), and reduced tumor proliferation (Ki-67). Transcriptomic profiling from these treated tumors revealed widespread downregulation of genes associated with TGF-β signaling, osteoblast differentiation, and bone remodeling [119]. Further evidence from multiple tumor models of prostate cancer confirms that antibody-mediated disruption of uPAR (e.g., huATN-658) attenuates metastatic growth, particularly skeletal metastasis, by interfering with uPAR–integrin interactions and downstream effectors such as FAK, AKT, and MAPK [120].

### 3.4. Regulation of Angiogenesis

Angiogenesis is critical for tumor growth and metastasis, supplying oxygen and nutrients to proliferating cancer cells. uPAR significantly contributes to this process via multiple, complementary mechanisms. Vascular endothelial growth factor (VEGF) stimulation upregulates uPAR expression and redistributes uPAR to the leading edge of migrating endothelial cells, where it co-localizes with integrin α_5_β_1_. This complex is internalized via LRP/clathrin-mediated endocytosis, concentrating localized proteolytic activity through the uPA/uPAR system, an essential step for endothelial migration and tube formation observed in in vivo angiogenesis models [110,121,122]. Blockade of this uPAR–integrin interaction impairs VEGF-induced endothelial migration both in vitro and in vivo, highlighting its functional importance [122].

Tumor-secreted suPAR also promotes angiogenesis. Conditioned media from tumor cells overexpressing uPAR enhance human umbilical vein endothelial cell (HUVEC) invasion, migration, and capillary-like tube formation. These activities are mediated via recruitment of suPAR to membrane lipid rafts, activation of ERK1/2 phosphorylation, and stimulation of Rac1 signaling, all critical for endothelial motility and angiogenic branching. Disruption of lipid rafts or blocking ERK/Rac1 signaling abolishes these effects, and in vivo tumor-bearing mouse models show elevated serum s-uPAR correlating with angiogenesis [123].

uPAR regulates angiogenesis by repressing PTEN expression in endothelial cells, thereby promoting endothelial survival and capillary formation. Silencing of uPA/uPAR in glioblastoma or endothelial models results in increased TIMP-1 secretion and soluble VEGF receptor-1 (sVEGFR1), inhibiting angiogenic sprouting [124]. Knockout or inhibition of uPAR abrogates VEGF-driven angiogenesis in mice, affirming the receptor’s centrality in this process [125]. Peptide-based uPAR inhibitors such as UPARANT, mimicking uPAR’s chemotactic sequence and antagonizing uPAR–integrin/GPCR interaction, effectively suppress angiogenesis in vivo and in vitro tumor models [126].

### 3.5. Modulation of the Tumor Microenvironment

The TME is a complex and dynamic ecosystem composed of cancer cells, stromal cells, immune cells, and ECM. uPAR plays a crucial role in shaping the TME to favor tumor growth and progression. Its overexpression in both cancer cells and stromal cells contributes to the creation of a pro-tumorigenic environment that supports cancer cell survival, proliferation, and immune evasion [127,128]. Within the TME, tumor-associated macrophages (TAMs) are key players in cancer progression. Macrophages can polarize into pro-tumorigenic M2 phenotypes, which secrete factors such as interleukin-6 (IL-6) and transforming growth factor-beta (TGF-β) [107,129,130]. These cytokines, influenced by uPAR, drive tumor growth, immune suppression, and angiogenesis, facilitating an environment conducive to metastasis [5,20,131]. In clinical studies, elevated uPAR expression in both tumor and stromal cells correlates with poor patient prognosis. For instance, high levels of uPAR in colorectal cancer patients have been linked to reduced overall survival and increased metastasis, highlighting the prognostic value of targeting uPAR within the TME [38].

In addition to its structural and signaling roles within the TME, uPAR significantly influences immune responses. It is broadly expressed on immune cells, including neutrophils, monocytes/macrophages, and T cells, where it regulates cell adhesion, migration, and activation through interactions with integrins and ECM components [24]. Pro-inflammatory cytokines such as IL-1β, TNF-α, and IL-6 are known to positively correlate with elevated uPAR expression and suPAR release under conditions of chronic inflammation and immune activation [24,132]. IL-2 stimulation has been shown experimentally to induce both uPA and uPAR expression in natural killer (NK) cells, enhancing their cytotoxic activity and motility [133]. Additionally, suPAR is elevated in various inflammatory diseases and cancers and serves as a robust biomarker of systemic immune activation and disease severity [42,134]. Altogether, these mechanisms underline uPAR’s critical role in orchestrating immune cell trafficking, activation, and inflammatory signaling that contribute to tumor progression and immune modulation within the TME.

## 4. Clinical Significance of uPAR as a Biomarker in Cancer

uPAR has garnered significant attention as a biomarker in cancer diagnostics and prognostics due to its overexpression in various malignancies [5,135,136]. Circulating suPAR is detectable in healthy adults at low nanogram-per-milliliter concentrations (assay-dependent reference interval; ELISA-based studies commonly report values in the ~2–3 ng/mL range) [137], whereas membrane uPAR is tissue/cell-surface expressed. Across inflammatory and malignant conditions, suPAR typically shifts upward, with cohort medians frequently higher than healthy baselines and upper tails extending further in severe disease [138,139].

### 4.1. Diagnostic and Prognostic Implications

uPAR expression levels have been correlated with tumor stage, grade, and patient prognosis across multiple cancer types. High levels of uPAR are often associated with more advanced stages of cancer, greater tumor invasiveness, and poorer overall survival rates [5,135]. For example, studies have shown that elevated uPAR expression in breast cancer patients is linked to higher rates of metastasis and reduced survival, making it a powerful prognostic marker for this disease [136,140,141].

In colorectal cancer, uPAR has been identified as a marker for early detection, with studies suggesting that its presence in tissue biopsies or in the blood can serve as an early warning sign of malignancy [38,142,143]. Moreover, in non-small cell lung cancer (NSCLC), uPAR levels have been used to stratify patients based on their risk of recurrence, aiding in the customization of treatment plans [144,145].

The ability of uPAR to predict treatment outcomes is another area of clinical significance. For instance, high uPAR expression has been linked to resistance to conventional therapies such as chemotherapy and radiotherapy [14,146,147,148]. This resistance is thought to be mediated through uPAR’s role in activating survival pathways like PI3K/AKT and MAPK/ERK, which help tumor cells evade apoptosis and continue proliferating despite treatment.

### 4.2. Therapeutic Targeting of uPAR in Cancer

**Figure 5:**
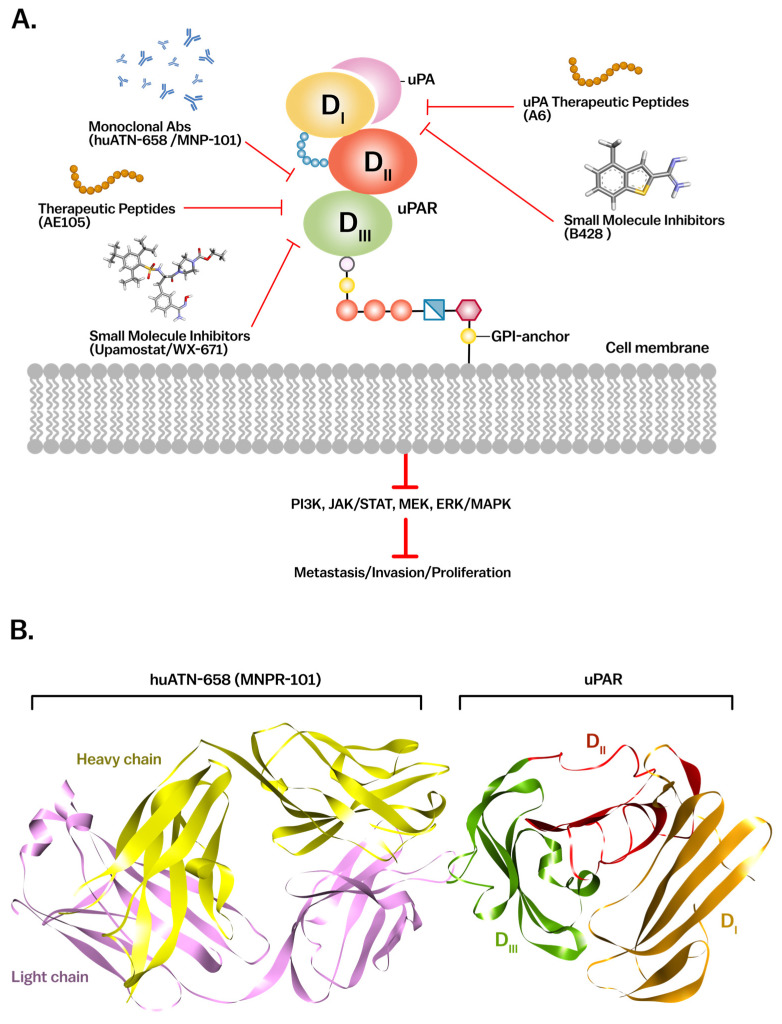
Therapeutic strategies targeting the uPA/uPAR system. (**A**) Cartoon summary of current therapeutic approaches targeting uPAR (left) and uPA (right), including monoclonal antibodies (e.g., huATN-658), therapeutic peptides (e.g., AE105, A6), and small molecule inhibitors (e.g., Upamostat, B428). These agents disrupt downstream signaling pathways (e.g., PI3K, JAK/STAT, MAPK) to inhibit tumor progression. (**B**) Crystal structure of huATN-658 (MNPR-101) in complex with uPAR, showing the antibody’s heavy (yellow) and light (purple) chains bound to uPAR (PDB ID: 4K24). mAb, monoclonal antibody; uPA, urokinase-type plasminogen activator; uPAR, urokinase-type plasminogen activator receptor; AE105/A6, uPA-derived peptides; B428/Upamostat (WX-671), small-molecule uPA inhibitors; PI3K, phosphoinositide 3-kinase; JAK/STAT, Janus kinase/signal transducer and activator of transcription; MEK, MAPK/ERK kinase; ERK/MAPK, extracellular signal-regulated kinase/mitogen-activated protein kinase; PDB, Protein Data Bank.

The therapeutic strategies targeting uPAR encompass a broad spectrum, including small molecule inhibitors, monoclonal antibodies, peptide-based inhibitors, RNA-based therapies, and novel combination therapies involving immune checkpoint inhibitors and other signaling pathways [2,45,58,149] (Figure 5A) (Table 2).

#### 4.2.1. Small Molecule and Peptide-Based Inhibitors

Small molecule inhibitors targeting the uPA-uPAR interaction have been among the earliest therapeutic approaches. One key example is Mesupron (Upamostat), a serine protease inhibitor that has shown promising results in Phase II clinical trials for metastatic breast and pancreatic cancers. Mesupron is the oral prodrug of WX-UK1. In HER2-negative metastatic breast cancer, mesupron + capecitabine showed a signal for benefit in predefined subgroups with a tolerable safety profile (mainly low-grade GI events/fatigue). In pancreatic cancer, mesupron + gemcitabine did not meet the primary survival endpoint overall, with comparable safety to gemcitabine; exploratory analyses suggested disease-control signals in subsets [150,151,152]. Another small molecule inhibitor, WX-UK1, which is a metabolite of Mesupron, has also demonstrated notable efficacy in preclinical models by reducing the invasiveness of various cancer cell types and inhibiting tumor growth through the blockade of uPA’s enzymatic activity [6]. These inhibitors work by disrupting the uPA-uPAR interaction, which is crucial for tumor metastasis and ECM degradation, making them promising agents in anti-cancer therapy [45,153]. Additionally, direct uPA inhibitors such as B428 have shown the ability to suppress uPA activity independent of receptor interaction, offering an alternative strategy to impair ECM degradation and tumor invasion. These inhibitors collectively disrupt the proteolytic cascade initiated by the uPA-uPAR axis, which is crucial for tumor metastasis and matrix remodeling, making them promising agents in anti-cancer therapy [154].

Peptide inhibitors are an important therapeutic avenue for targeting uPAR’s role in tumor progression. One key example is the amino-terminal fragment (ATF) of uPA, which has been used to create competitive inhibitors that block the interaction between uPA and uPAR. These inhibitors prevent downstream signaling pathways that are critical for tumor cell migration and metastasis. The ATF’s ability to bind uPAR without activating its proteolytic functions makes it a promising therapeutic strategy for targeting cancers dependent on uPAR activity [117,155].

Another peptide-based inhibitor, the A6 peptide, a non-enzymatic fragment derived from uPA’s connecting domain, has shown anti-metastatic and anti-angiogenic activity across several cancer types by interfering with uPA-mediated cell signaling. Unlike ATF, A6 does not block uPA binding directly but exerts its effects by modulating cell adhesion and migration pathways [156,157].

A notable peptide inhibitor, AE105, has been radiolabeled and used both as a therapeutic agent and a diagnostic tool for imaging uPAR expression in tumors. This peptide binds with high affinity to uPAR and has been utilized in PET imaging to visualize uPAR-positive tumors in models such as prostate and glioblastoma. AE105 also inhibits uPAR’s role in cancer cell invasion, making it a dual-purpose tool for both diagnosis and treatment [117,155,158].

#### 4.2.2. Monoclonal Antibodies

Monoclonal antibodies targeting uPAR represent a significant advancement in cancer therapy by disrupting key interactions involved in tumor progression and metastasis. One of the most promising antibodies, huATN-658 (also known as MNPR-101 recently acquired by Monopar Therapeutics), has shown exceptional efficacy in preclinical models of prostate, breast, and ovarian cancers. Unlike other uPAR-targeting strategies, huATN-658 does not block the binding of uPA but instead disrupts downstream signaling, inhibiting tumor cell migration, invasion, and proliferation [159,160] (Figure 5B). This monoclonal antibody has demonstrated robust anti-tumor activity in a broad range of cancers, making it a leading candidate for clinical trials. In addition, 2G10, another monoclonal antibody, targets the interaction between uPAR and integrins, inhibiting tumor cell adhesion and invasion [160]. This approach is particularly effective in preventing metastasis by blocking the cellular mechanisms that allow tumor cells to invade surrounding tissues [161,162]. These monoclonal antibodies, when combined with traditional chemotherapy or immune checkpoint inhibitors, represent novel approaches to targeting tumor progression. Early clinical data for MNPR-101 suggests its potential for broad therapeutic application in uPAR-expressing tumors [160].

#### 4.2.3. RNA-Based Therapies

RNA interference (RNAi) has been employed to silence uPAR expression in cancer cells. siRNA and shRNA targeting uPAR mRNA have demonstrated the ability to reduce uPAR levels, leading to decreased tumor cell invasion, migration, and growth in various preclinical models [163,164]. These RNA-based approaches hold promise for personalized cancer therapy, particularly when integrated with other treatment modalities [165,166,167].

#### 4.2.4. Combination Therapies Involving Immune Checkpoint Inhibitors

Advances in immunotherapy have driven the exploration of uPAR-targeting agents in combination with immune checkpoint inhibitors (ICIs) such as anti-PD-1 antibodies. The rationale is that uPAR activity fosters a myeloid-driven, immunosuppressive tumor microenvironment that limits cytotoxic T-cell access; inhibiting uPAR should relieve this brake and improve checkpoint efficacy [168]. This combination leverages uPAR’s role in promoting an immunosuppressive tumor microenvironment. By blocking uPAR, these therapies help reduce tumor-associated immunosuppression, enhancing the efficacy of PD-1 inhibitors. Emerging preclinical models have demonstrated encouraging responses when uPAR inhibitors are combined with PD-1 blockade, including enhanced T-cell infiltration and tumor regression [169]. This combinatorial approach shows potential in overcoming resistance mechanisms associated with monotherapy and may help broaden the clinical applicability of immune checkpoint inhibitors, particularly in tumors with high uPAR expression [170,171]. These findings are significant because uPAR expression is associated with high infiltration of immunosuppressive cells, such as regulatory T cells (Tregs) and myeloid-derived suppressor cells (MDSCs) [172]. Targeting uPAR helps diminish this suppression, enabling immune checkpoint inhibitors to elicit stronger T-cell-mediated responses. The dual inhibition of uPAR and PD-1 not only enhances the immune response but also contributes to a better long-term anti-tumor effect, reducing tumor relapse and metastasis [169,173]. Although further validation is needed, early findings suggest that targeting uPAR may synergize with ICIs to amplify anti-tumor immunity and improve long-term outcomes. This represents a promising direction for the development of next-generation immunotherapy strategies.

#### 4.2.5. Role of PAI-1 in uPAR-Targeted Therapy

Plasminogen activator inhibitor-1 (PAI-1) is a critical regulator of the uPA-uPAR system, and its role in cancer biology has been extensively studied. While PAI-1 inhibits uPA activity, paradoxically, high levels of PAI-1 are often associated with poor prognosis in cancer patients. This is due to PAI-1’s ability to stabilize the uPA-uPAR complex on the cell surface via LRP1-mediated endocytosis/recycling [174], promoting cell adhesion, migration, and angiogenesis. Therapeutic strategies targeting uPAR often consider the interplay between uPAR and PAI-1, with some approaches aiming to modulate PAI-1 activity to enhance the efficacy of uPAR-targeted treatments. For instance, dual targeting of uPAR and PAI-1 has been proposed to disrupt the uPA-uPAR-PAI-1 axis more effectively, thereby inhibiting tumor progression and metastasis [93,175].

Despite significant progress, several challenges persist in uPAR-targeted therapy. One major challenge is the expression of uPAR in normal tissues, which can lead to off-target effects and toxicity. Moreover, the complexity of uPAR expression within tumors and across different patients complicates the development of universally effective therapies. To address these challenges, research is ongoing to develop more selective inhibitors with higher affinity for uPAR, as well as to optimize combination therapies that target multiple pathways involved in tumor growth and metastasis [10]. Nanotechnology-based delivery systems are also being explored to enhance the targeted delivery of uPAR inhibitors to tumor sites, thereby reducing systemic toxicity and improving therapeutic outcomes [2,10,176].

**Table 2:** List of Therapeutic Approaches Targeting uPAR in Cancer Treatment.

Therapeutic Approach	Therapeutic Agents	Mechanism of Action	References
Monoclonal Antibodies (mAbs)	huATN-658-Humanized mAb targeting uPAR DII–DIII (MNPR-101)	Blocks uPAR-uPA interaction, reducing tumor growth and metastasis	[149,159,177,178]
	2G10-Anti-uPAR mAb	Inhibits uPAR-α5β1 integrin interaction, reducing migration and invasion	[161,162,162,179]
	VH Domains Targeting uPAR	Human antibody VH domains targeting uPAR for reducing metastasis and tumor growth	[180]
	4G10-Anti-uPAR mAb	Targets uPAR-integrin interaction, reducing metastasis	[181,182]
	8B12	Blocks uPA-uPAR interaction, reducing ECM degradation and cancer cell invasion	[182,183,184]
Small Molecule Inhibitors	Upamostat (WX-671)-Oral uPAR inhibitor	Blocks uPA binding to uPAR, inhibiting plasminogen activation and tumor invasion	[45,153,185]
	IPR-456	Inhibits uPAR, blocking cell invasion and metastasis	[186]
	IPR-803	Inhibits uPAR-mediated signaling, reducing tumor cell invasion and metastasis	[187,188]
	ARM-U2	Inhibits uPAR-uPA interaction, reducing metastasis and tumor growth	[189]
	LLL fsi	Blocks uPAR signaling, reducing cancer cell invasion and metastasis	[190]
	Compound **6**	Blocks cell adhesion to Vn Block cell adhesion to vitronectin and impair FPR-dependent cell migration	[191]
	Compound **37**		
	2-(Pyridin-2-ylamino)-quinolin-8-ol	Inhibits uPAR, impairing cell migration and reducing metastasis	[105]
	2,2′-(methylimino)di (8-quinolinol)	Inhibits uPAR, blocking cell migration and tumor progression	
Therapeutic Peptides	WX-360	Inhibits uPAR, reducing tumor cell migration and invasion	[192,193]
	AE105	Binds to uPAR, blocking its interaction with ligands, reducing tumor invasion	[194]
	AE120	Targets uPAR, inhibiting cancer cell migration and metastasis	
	pERERY-NH2	Interferes with uPAR-mediated signaling, impairing tumor cell invasion	[195]
	RERF	Inhibits uPAR, blocking cancer cell adhesion and migration	[196]
	UPARANT (cenupatide)	Blocks uPAR activation, reducing tumor angiogenesis and metastasis	[192,193,194,195,196,197,198,199,200,201,202,203]
	RI-3	Inhibits uPAR-uPA interaction, impairing cancer progression	[204]
	Cyclized SRSRY	Inhibits uPAR, blocking integrin interaction, reducing cell migration	[205]
	SRS(P)RY	Disrupts uPAR signaling, impairing cell migration and invasion	[206]
	P25	Binds uPAR, blocking interactions with ligands, reducing metastasis	[68,207]
	M25	Impairs β1-integrindependent spreading and migration	[208]
	α325	Blocks filopodia formation and matrix invasion in vitro	[209,210]
	m.P243-251	Binds to uPAR, impairing cancer cell invasion and metastasis	[211]
Nanodrugs	AE147 Peptide-Conjugated Nanocarriers	Targets uPAR-overexpressing cancer cells, delivering therapeutic agents and reducing metastasis	[176,212]

## 5. uPAR in Other Diseases

Initially recognized for its involvement in oncology, uPAR has since been implicated in a wide array of diseases, including cardiovascular diseases, infectious diseases, and neurological disorders [25,40] (Table 3). uPAR chiefly amplifies plaque inflammation and remodeling (monocyte/smooth muscle cells (SMC) recruitment, ECM degradation) with thrombotic consequences; suPAR is prognostic but influenced by systemic inflammation and renal status. In infectious disease, uPAR primarily modulates host defense and sepsis biology (neutrophil/monocyte trafficking, cytokine amplification) and serves as a severity/risk stratifier; post-viral fibrosis links uPAR to TGF-β–driven remodeling. In the central nervous system, uPAR is tied to blood–brain barrier disruption, microglial activation, and demyelination, shifting therapeutic focus toward neuroinflammation/BBB stabilization rather than antithrombotic strategies. These distinctions frame the mechanism and clinical implications detailed in Section 5.1, Section 5.2 and Section 5.3.

### 5.1. uPAR in Cardiovascular Diseases

Atherosclerosis is a chronic inflammatory condition characterized by the buildup of plaques within the arterial walls, consisting primarily of lipids, cholesterol, and immune cells [213]. uPAR, through its interaction with uPA, facilitates the migration of smooth muscle cells (SMCs) and monocytes/macrophages into the vascular intima, which accelerates plaque formation and progression [214]. Elevated uPAR expression is observed within atherosclerotic lesions, particularly in vulnerable plaques that are prone to rupture [215].

The presence of uPAR in macrophages within plaques enhances the proteolytic degradation of the ECM, contributing to plaque destabilization and increasing the risk of plaque rupture [216]. When a plaque ruptures, the pro-thrombotic content within the lesion is exposed to circulating blood, initiating the clotting cascade and leading to thrombosis, the most common cause of acute coronary syndrome (ACS) and myocardial infarction (MI) [217]. uPAR also plays a key role in post-MI cardiac remodeling, where inflammation and ECM degradation lead to adverse structural changes in the myocardium [218]. Elevated levels of suPAR have been proposed as a biomarker for cardiovascular events and predict mortality in patients with CVDs [214].

Studies have linked high suPAR levels with increased systemic inflammation, which is a major contributor to the pathogenesis of atherosclerosis [219,220,221,222,223]. Inhibition of uPAR and the modulation of the uPA-uPAR axis have been explored as potential therapeutic strategies to prevent plaque formation and reduce the risk of cardiovascular events.

### 5.2. uPAR in Infectious Diseases

uPAR plays a crucial role in the body’s response to both bacterial and viral infections, influencing the balance between immune protection and excessive inflammation [224]. During bacterial infections, such as those caused by *Streptococcus pneumoniae* or *Escherichia coli*, uPAR enhances the recruitment of immune cells to sites of infection, particularly neutrophils and monocytes, facilitating bacterial clearance [225]. However, this heightened immune response can also lead to tissue damage, especially in cases of sepsis, where an overactive immune response can cause widespread inflammation and organ failure.

In the context of viral infections, including HIV and SARS-CoV-2 (COVID-19), elevated uPAR expression has been associated with worse clinical outcomes. For instance, in COVID-19, high suPAR levels are linked to severe disease and poor prognosis, likely due to the role of suPAR in enhancing systemic inflammation and immune dysregulation [226,227]. suPAR has been explored as a biomarker for identifying patients at higher risk of severe disease, guiding therapeutic interventions [24,228,229].

uPAR is implicated in the pathogenesis of lung fibrosis, particularly in the setting of chronic inflammation and viral injury such as that seen in severe COVID-19 [230]. Persistent activation of the uPA–uPAR axis contributes to ECM remodeling, excessive fibroblast activation, and impaired resolution of inflammation, hallmarks of fibrotic lung disease. In COVID-19, elevated levels of suPAR have been correlated with both acute respiratory distress syndrome (ARDS) and the subsequent development of interstitial lung fibrosis [231]. suPAR promotes the recruitment of immune cells and enhances profibrotic cytokine signaling, such as TGF-β, which drives myofibroblast differentiation and collagen deposition. Experimental studies and patient data have shown that high uPAR activity is associated with reduced lung function, increased ECM stiffness, and progressive fibrotic remodeling, even after viral clearance [232,233]. These findings position uPAR as a potential biomarker for fibrotic progression and a candidate target for antifibrotic therapies, not only in COVID-19 but also in idiopathic pulmonary fibrosis (IPF) and post-viral fibrotic syndromes.

uPAR also modulates the innate immune response by regulating macrophage and T-cell activity [22,42]. By influencing the activation and migration of immune cells, uPAR contributes to the overall immune response against infections, while its excessive activity can promote inflammation and tissue damage, as observed in chronic infections and severe viral diseases [98].

### 5.3. uPAR in Neurological Disorders

uPAR has been implicated in both neurodegeneration and developmental disorders. Its expression is upregulated in the brain during conditions such as Alzheimer’s disease (AD), Parkinson’s disease (PD), and multiple sclerosis (MS) [234,235,236,237]. In AD, uPAR facilitates the breakdown of the blood–brain barrier (BBB), promoting the infiltration of inflammatory cells into the brain [238]. This contributes to chronic neuroinflammation, which exacerbates the pathological features of the disease, such as amyloid-beta deposition and tau protein aggregation [239].

uPAR also plays a role in multiple sclerosis, where its expression is increased in T cells and microglia within the central nervous system (CNS). This enhances the migration of these immune cells across the BBB, driving the inflammatory processes that lead to demyelination and neuronal damage [240]. Additionally, uPAR is involved in matrix degradation in the brain, disrupting the ECM and contributing to the loss of neuronal support and function [241].

Moreover, the interaction of uPAR with uPA and other proteases in the CNS leads to the degradation of the ECM, further contributing to neurodegenerative processes [242]. Therapeutic strategies targeting uPAR in neurological disorders focus on modulating its role in inflammation and ECM degradation to mitigate neurodegeneration and protect neuronal integrity [243]. uPAR is also implicated in neurodevelopmental disorders. Functional polymorphisms in the *PLAUR* gene have been found to increase the risk of autism spectrum disorder (ASD) [244]. Additionally, studies on uPAR knockout mice have revealed that they exhibit enhanced sensitivity to pharmacologically induced seizures, increased anxiety, and impaired social behavior [245]. These findings strongly suggest the significant role of uPAR in neurodevelopmental conditions and warrant further investigation.

**Table 3:** uPAR Across a Spectrum of Diseases: Role, Mechanisms, and Implications.

Disease Category	Role of uPAR	Mechanism/Process Involved	Implications	References
Cardiovascular Diseases	Atherosclerosis, Thrombosis, Cardiac Remodeling	uPAR promotes smooth muscle cell migration, ECM degradation and inflammation	Increases risk of cardiovascular events (e.g., MI, heart failure)	[40,122,246]
Infectious Diseases	Bacterial Infections (e.g., *Streptococcus*), Viral Infections (e.g., COVID-19)	uPAR regulates immune cell recruitment; suPAR elevated in severe cases	Biomarker for disease severity, impacts infection outcomes	[16,226,247,248,249]
Lung Fibrosis	Promotes fibroblast activation, immune cell recruitment, and ECM remodeling	uPAR/uPA axis drives TGF-β signaling, myofibroblast differentiation, collagen deposition, and chronic inflammation	Contributes to ARDS, post-viral fibrosis (e.g., COVID-19), and idiopathic pulmonary fibrosis (IPF)	[230,231,232,233]
Neurological Disorders	Neurodegeneration (e.g., Alzheimer’s, Parkinson’s), MS	uPAR mediates BBB breakdown, ECM degradation, neuroinflammation	Contributes to neurodegeneration, demyelination, cognitive decline	[18,250,251,252,253]
Autoimmune Diseases	Rheumatoid Arthritis, Lupus	uPAR involved in joint inflammation, ECM remodeling, immune response	Promotes chronic inflammation, joint destruction	[254,255,256,257,258]
Ocular Diseases	Age-Related Macular Degeneration (AMD)	uPAR involved in abnormal blood vessel growth (neovascularization)	Linked to progression of AMD and other retinal diseases	[259,260]
Pulmonary Diseases	Chronic Obstructive Pulmonary Disease (COPD), Asthma	uPAR involved in airway remodeling and inflammation	Correlates with disease severity and lung function decline	[44,261,262]
Renal Diseases	Glomerulonephritis, Chronic Kidney Disease (CKD)	uPAR regulates podocyte dysfunction, immune responses	Associated with kidney damage, proteinuria	[263,264,265]
Gastrointestinal Diseases	Inflammatory Bowel Disease (IBD), Crohn’s Disease	uPAR promotes mucosal inflammation, immune cell migration	Associated with worsened inflammation and disease severity	[265,266,267]
Cancer Metastasis	Various Cancers	uPAR mediates ECM degradation, cell migration, invasion	Associated with cancer progression, metastasis	[34,36,268,269,270,271]

## 6. Novel Insights into uPAR Function and Regulation

uPAR expression is modulated by epigenetic changes, including DNA methylation and histone modifications. Hypomethylation of the *PLAUR* gene promoter has been linked to increased uPAR expression in cancers such as colorectal and breast cancers, where it correlates with enhanced metastatic potential and poor prognosis [272,273,274,275]. Epigenetic modifications not only drive uPAR overexpression but also contribute to tumor aggressiveness by facilitating processes like invasion and angiogenesis.

Recent findings underscore the role of miRNAs in regulating uPAR expression at the post-transcriptional level. miRNAs such as miR-193b and miR-23b specifically target uPAR mRNA, suppressing its translation and inhibiting tumor invasion and metastasis. The downregulation of these miRNAs is often associated with increased uPAR levels, contributing to a more invasive tumor phenotype [276,277,278,279,280].

Post-translational modifications, particularly glycosylation, are critical for uPAR’s structural stability and its interactions with extracellular matrix proteins like vitronectin and integrins. Glycosylation ensures proper cell surface localization, allowing uPAR to mediate cell adhesion and migration [51,281]. Additionally, proteolytic cleavage of uPAR generates suPAR, which retains its activity and serves as a biomarker in various cancers and inflammatory conditions as discussed previously. Although uPAR itself does not undergo phosphorylation, it triggers phosphorylation cascades in downstream signaling pathways through interactions with integrins and receptors like EGFR. For instance, uPAR’s binding to α5β1 integrin activates the phosphorylation of FAK at Tyr397, initiating the Ras/MAPK pathway crucial for cell migration and proliferation [2]. Furthermore, uPAR indirectly regulates the phosphorylation of key proteins such as AKT, ERK, and mTOR, enhancing cell survival and tumor progression [282,283].

## 7. Conclusions and Future Perspectives

While considerable progress has been made in understanding uPAR’s role in various diseases, there remain notable gaps in our knowledge that warrant further investigation [99,283,284]. Although its involvement in processes like proteolysis and cell migration has been well characterized, its influence on immune regulation and matrix remodeling in non-oncological diseases, such as chronic inflammation and neurodegeneration, remains unclear [285]. Moreover, there is debate over the long-term viability of uPAR-targeting therapies, particularly concerning their efficacy in highly heterogeneous and treatment-resistant tumors [286,287]. The role of suPAR as a biomarker in diverse diseases is another area of controversy, with conflicting data on its predictive value across conditions such as cardiovascular disease and chronic infections [42,288,289]. Going forward, three priorities can convert biology into impact: harmonize suPAR assays with indication-specific cut-offs; deploy companion diagnostics (uPAR-PET and quantitative tissue scoring) for patient selection and pharmacodynamic readouts; and advance uPAR-directed agents in rational combinations while mapping on-target/off-tumor safety.

Cutting-edge technologies such as single-cell RNA sequencing and spatial transcriptomics have begun to revolutionize our understanding of uPAR’s roles in disease [290]. Single-cell RNA-seq from 37 glioblastoma patients identified *PLAUR* (uPAR) as a marker for two intra-tumoral subtypes, one enriched for inflammatory programs and the other for ECM-remodeling/invasive programs, and mapped co-expressed partners (e.g., CD44, and FN1) without presupposing pathways, highlighting where uPAR couples immunity and invasion in situ [290]. These advanced methodologies enable a higher-resolution analysis of uPAR expression in various cell types and tissue environments, revealing functional heterogeneity that was previously undetectable. These tools have been particularly effective in dissecting cellular interactions within the tumor microenvironment, providing clearer insights into the role of uPAR in tumor progression, immune modulation, and metastasis [290]. One key area of emerging research is uPAR’s involvement in immune modulation, particularly within the tumor microenvironment [107,110,291]. uPAR appears to facilitate immune evasion, promoting tumor survival and resistance to therapies. Escape to uPAR-directed therapy can arise via protease redundancy (e.g., MMP-2/-9 and cathepsins) and uPAR–integrin/FAK–SRC → PI3K–AKT pathway rewiring, as well as PAI-1–LRP1-mediated uPAR recycling that restores pericellular proteolysis. Countermeasures include patient selection with companion diagnostics and rational combinations alongside uPAR targeting [292]. Additionally, new hypotheses suggest that uPAR may be involved in epigenetic regulation, interacting with non-coding RNAs to modulate gene expression and tumor behavior, thereby offering new potential targets for therapy [272,293,294].

Beyond oncology, uPAR’s relevance extends to select cardiovascular [295,296,297] and neurological conditions; for mechanistic detail and evidence synthesis, see Section 5 [242,298,299].

Agents such as MNPR-101 (formerly ATN-658), an anti-uPAR antibody currently in phase 1 clinical trials (https://clinicaltrials.gov/study/NCT06617169 (accessed on 8 October 2025)), represent a major leap forward in precision oncology [160,300].

uPAR-targeted imaging techniques, such as PET using radiolabeled ligands, are proving invaluable for real-time tracking of disease progression in cancer and cardiovascular diseases. These tools not only improve diagnostic accuracy but also enable the monitoring of therapeutic responses in a minimally invasive manner [160,301,302,303].

As our understanding of uPAR’s role in disease mechanisms continues to evolve, particularly through the use of advanced molecular techniques, we will likely see the emergence of more refined diagnostic tools and treatment options. The integration of uPAR research into broader multi-omics platforms will enhance the precision of predictive models, enabling earlier interventions and improving patient outcomes across multiple medical disciplines [304].

## Data Availability

No new data were created or analyzed in this study.
